# A novel two-step sequential bioaccessibility test for potentially toxic elements in inhaled particulate matter transported into the gastrointestinal tract by mucociliary clearance

**DOI:** 10.1007/s00216-017-0257-2

**Published:** 2017-02-24

**Authors:** Jawad Ali Hussein Alpofead, Christine M. Davidson, David Littlejohn

**Affiliations:** WestCHEM, Department of Pure and Applied Chemistry, University of Strathclyde, 295 Cathedral Street, Glasgow, G1 1XL UK

**Keywords:** Bioaccessibility, Inhaled particulate matter, Potentially toxic elements, Artificial mucus fluid

## Abstract

A novel two-step sequential extraction has been developed to assess the bioaccessibility of As, Cd, Cr, Cu, Fe, Mn, Ni, Pb and Zn in airborne particulate matter following inhalation and transport into the human gastrointestinal tract by mucociliary clearance. A new artificial mucus fluid (AMF) was used to determine the bioaccessible potentially toxic element (PTE) fraction in the upper airways, in sequence with the simplified bioaccessibility extraction test (SBET) or the stomach phase of the unified bioaccessibility method (gastric fluid only) (UBMG). Filter dynamic measurement system TX40 filters smeared with soil reference material (BGS RM 102) were used as test samples. Analysis was performed by ICP-MS. Comparison between results obtained for soil alone and when the soil was supported on TX40 filters indicated that the presence of the substrate did not affect the extraction efficiency, although a large Zn blank was detected. The sequential AMF→SBET extraction liberated similar amounts of Fe, Mn, Ni and Zn to the SBET alone; but significantly less Cd; and significantly more As, Cr, Cu and Pb. The sequential AMF→UBMG extraction liberated similar amounts of Cd, Cr, Mn and Zn to the UBMG alone, but significantly less As, Fe and Ni; and significantly more Cu and Pb. Enhanced extractability was due to the greater quantities of exchangeable ions and complexing agents present. Adoption of a two-step sequential extraction (AMF followed by either the SBET or the UBMG) is recommended because it is more representative of biological conditions and avoids overestimation or underestimation of bioaccessible PTE concentrations.

**Graphical Abstract:**
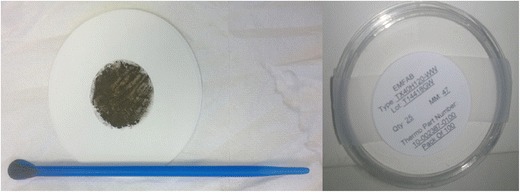
Simulated PM_10_ sample: BGS RM 102 ironstone soil on TX40 filter

Simulated PM_10_ sample: BGS RM 102 ironstone soil on TX40 filter

## Introduction

Potentially toxic elements (PTE) associated with inhalable airborne particulate matter (PM_10_) can cause adverse effects on human health, such as increased prevalence of heart disease and lung cancer, especially in urban and (current and former) industrial areas [1, 2]. The severity of health effects depends on the availability of the PTE for absorption into the body, and this has led to growing interest in the development of analytical protocols to estimate the bioaccessibility of PTE bound to PM_10_ following inhalation of contaminated particles [3].

The human respiratory system consists of two functional regions: the conducting airways (i.e. the nose, pharynx, larynx, trachea, bronchi and bronchioles) and the respiratory region (i.e. the lungs) [1, 4, 5]. Airways surface liquid (ASL) lines the conducting regions [2, 6]. This consists of three layers: a basal sol layer, a thin layer of surfactant, and a mucus layer. The viscosity and elasticity of the mucus layer are higher than the sol layer, and so the mucus can be cleared out of the deeper airways and nasal cavity, and transported into the gastrointestinal tract by movement of cilia [1, 7].

Inhaled PM is initially trapped by the mucus layer [1, 7]. Thereafter, particles <2.5 μm in diameter penetrate to the lungs [2], whilst particles with diameter in the range 2.5–10 μm (which can constitute up to 95% of those inhaled [8]) are transported from the conducting airways to the gastrointestinal tract by mucociliary clearance [2, 9, 10]. Leaching can occur during transport and substances released can be absorbed by cells lining the conducting airways [2, 9, 11–13]. Thus, although the mucus layer is sometimes considered one of the barriers to absorption of inhaled substances in the respiratory system, it can also serve as a transport pathway by which PTE associated with PM can enter the body, with factors such as molecular weight, solubility and electrical charge determining the absorption kinetics for specific species [2]. As PTE can dissolve and be absorbed both *en route* to, and within, the stomach, analytical methods used to assess the bioaccessibility of PTE associated with inhaled PM should ideally include two sequential compartments: the first representing the conducting airways and the second representing the gastrointestinal tract. To date, however, only single step extractions have been applied in this field.

The original [14] and modified [3, 15–20] versions of Gamble’s solution have been used in studies to estimate the inhaled bioaccessible fraction of PTE in PM_10_ derived from a variety of sources, including coal-derived fly ash [21], mine waste [18, 22], urban surface soils [39] and smelters dust. [23] In addition, PM_10_ collected on different types of filters (quartz fibre filters [24, 25], Teflon filters [26] and cellulose nitrate filters [27]) have been analysed. A key study [3] proposed a novel in vitro simulated epithelial lung fluid that contained high molecular mass proteins, antioxidants and a surfactant, in addition to inorganic components, and so more closely simulated conditions in the real human respiratory system. This was successfully applied to assess the bioaccessibility of Pb following inhalation of PM_10_ from urban surface soils, tailings and smelter wastes from Mitrovica, Kosovo. The work clearly demonstrated the importance of the direct inhalation pathway in adding significantly to Pb intake for a population chronically exposed to contaminated dust. However, the fluid used represented the sum of the two layers of ASL, whereas PM_10_ are generally in contact only with mucus. Developments of a specifically mucus-like extractant—an artificial mucus fluid (AMF)—would therefore be desirable.

For the second compartment, any established oral bioaccessibility extraction test could in principle be used. Both the simplified bioaccessibility extraction test (SBET) [28] and the unified bioaccessibility method (UBM) [29] have been employed for determining the bioaccessible fraction of PTE in PM [30–34]. In some cases, the PM_10_ was obtained from urban soil samples [30, 31] or from urban street dust [32] whilst, in others, filter-based samples were used [33, 34]. A recent study [34] miniaturised the SBET and the stomach phase of the UBM, and successfully applied the methods developed to measure the bioaccessible fraction of PTE in PM_10_ supported on TX40 filters employed in the filter dynamics measurement system (FDMS) samplers widely used for continuous monitoring of ambient particulate matter.

The aim of the current study was to establish a two-step sequential extraction method for determining the bioaccessible fraction of PTE in PM_10_ transported to the gastrointestinal tract by mucociliary clearance that more accurately simulates biological conditions. The procedure developed incorporated the following:

- A novel AMF to assess bioaccessibility during transport, followed by
- A modified SBET or a stomach phase UBM test [34] to assess bioaccessibility on arrival in the stomach.

As in previous work [34], the study was carried out using surrogate PM_10_ samples prepared from a soil reference material—BGS RM 102 Ironstone Soil (with target values for amounts of As, Cd and Pb extractable by the UBM)—since this allowed replicate test samples to be prepared for use in method comparison and to assess the repeatability of the new extraction. The surrogate samples were prepared on TX40 filters so that any issues arising from extracting particles supported on filters could be detected and overcome (e.g. the presence of filter blanks) with the goal of developing an analytical method that would be directly applicable to genuine PM samples collected as part of routine air quality monitoring campaigns worldwide.

## Experimental

### Selection of constituents for AMF and AMF extraction parameters

Table 1 shows the composition of the AMF used in step 1 of the sequential extraction of simulated PM_10_ samples. Mucus is a heterogeneous mixture of proteins, lipids and salts [9, 11, 35]. After water, which constitutes 95–97% [7] of mucus, the second most abundant components by mass are proteins. These include mucins—glycoproteins responsible for the viscoelastic properties of mucus [35]—and serum proteins such as albumin [7, 9, 11, 35]. Mucus is typically composed of 0.5–1% free protein and 0.5–2% mucin [1, 4, 11]. Due to low solubility in water, and to minimise the total dissolved solids content in extracts, a mucin concentration of 0.5% (i.e. 5 g L^−1^) was selected. The concentration of albumin in ASL is 480–730 mg L^−1^ [4] and the middle of this range (610 mg L^−1^) was used. Inorganic salts constitute ∼1% by weight of mucus [1, 7, 11, 27]; the concentrations of Na^+^, Cl^−^ and K^+^ in ASL have been reported as 1838–1953, 2658–2836 and 586 mg L^−1^, respectively [4], or approximately 45% less than plasma for Na^+^ and Cl^−^ and 600% more than plasma for K^+^ [11]. The concentrations of KCl, NaCl and NaHCO_3_ in AMF were chosen based on the above values, whilst other salts, i.e. NaH_2_PO_4_, Na_2_SO_4_, CaCl_2_·2H_2_O and MgCl_2_·6H_2_O, were incorporated at levels similar to their concentrations in the original Gamble solution [14]. Lipids are another of the principle components of mucus [7, 9, 11, 35]; it is composed of 1% lipids [1] that reduce the surface tension between the layers of ASL [1, 9]. The majority of mucus lipids are phospholipids, and the most abundant is phosphatidylcholine [4], which constitutes 11% of total lipids (i.e. 0.11% of mucus by mass) [36]. Therefore, 1.10 g L^−1^ of dipalmitoyl phosphatidylcholine (DPPC) was included in the AMF. Besides mucins, secretory cells release a variety of antimicrobial molecules, e.g. lysozyme [7, 11], that destroys bacteria in respiratory mucus [35]. The concentration of lysozyme in ASL is 0.1–1 mg mL^−1^ [4] and the middle of this range (0.5 mg mL^−1^) was chosen. Glutathione is also present in ASL at 132 mg L^−1^ [4] and so this concentration was adopted.

**Table 1:** The composition of artificial mucus fluid (AMF)

Reagent	Weight of reagent (mg) made up to 100 mL with deionised water
Inorganic reagent	
KCl	224
NaH_2_PO_4_	24
Na_2_SO_4_	14
NaCl	620
CaCl_2_·2H_2_O	74
NaHCO_3_	504
MgCl_2_·6H_2_O	42
Organic reagent	
Glutathione	26.4
Additional reagents	
Mucin	1000
Albumin	122
DPPC	220
Lysozyme	100

*DPPC* dipalmitoyl phosphatidylcholine

According to Sexton et al. [37], a moderate seasonal inhalation dose for PM_10_ is 100 mg and so this sample mass was selected. Human studies using the tablet inhalation technique [38] demonstrated that all deposited particles >6 μm in diameter were removed from the airways by mucociliary clearance within 24 h and that 49 ± 9% of particles had a mean half time of 3.0 ± 1.6 h in healthy circumstances. Given that the average lengths of the human pharynx, larynx, trachea, right and left bronchus are 13, 10.4, 12, 2.5 and 5 cm, respectively [39], and that minimum and maximum velocities of mucociliary clearance are 0.4 and 2 cm min^−1^ [1, 4, 11], an extraction time of 1 h was selected. The maximum volume of ASL produced daily ranges from 100 mL [4] to >125 mL [40]. Estimates of the relative proportions that are absorbed and transported to the gastrointestinal tract differ: Schans [12] reported that 20 mL of mucus per day reached the trachea, whilst King [9] found that 10 mL per day reached the larynx, and Fahy [7] claimed 30 mL per day was deposited in the gastrointestinal tract. In the current study, 120 mL was chosen for the volume of mucus produced per day and 30 mL per day for the volume transported to the stomach by mucociliary clearance. Hence, the AMF extraction was performed for 1 h using 5 mL of reagent, 1.5 mL of which was carried forward into step 2—the gastrointestinal step—of the sequential extraction. Overall, ASL is very slightly acidic (mean pH 6.78 [4, 6]), but the pH of mucus is around 7 [41] and so the latter value was selected for the AMF extraction. As the extraction was intended to simulate physiological conditions, it was carried out at the basal human body temperature. The final protocol thus involved extraction of 100 mg samples for 1 h in 5 mL of AMF adjusted to a pH of 7.00 ± 0.20 at 37 ± 2 °C (for further details, see “Preparation of AMF” and “Sequential bioaccessibility extraction procedure” sections).

### Apparatus and reagents

Blank Pallflex TX40 FDMS filters were supplied by Air Monitors (Gloucestershire, UK). These are made of borosilicate microfibres reinforced with woven glass cloth and bonded with polytetrafluoroethylene (5 mg cm^−2^, 47 mm diameter). The pH of solutions was measured by using a Mettler-Teledo (SevenGo™) pH meter. Suspensions were shaken in an end-over-end rotator inside an incubator (Stuart® SI500 shaking incubator from Barloworld Scientific Ltd., Staffordshire, UK). All glassware and plastic ware were soaked overnight in 10% HNO_3_ then rinsed three times with deionised water before use. All chemicals were of analytical grade. Bovine serum albumin, NaH_2_PO_4_, KCl, pancreatin (porcine), urea, CaCl_2_·2H_2_O and pepsin (porcine) were purchased form Merck (Poole, UK). Glucose, NaCl, Na_2_SO_4_, NH_4_Cl, NaHCO_3_ and NaOH were supplied by VWR International (Lutterworth, UK). Lysozyme, glutathione, MgCl_2_·6H_2_O, glucuronic acid, glucosamine hydrochloride, mucin (porcine), DPPC, hydrochloric acid (HCl) (36.5–38%) and nitric acid (HNO_3_) (≥69% Trace SELECT® for trace analysis) were obtained from Sigma Aldrich (Gillingham, Dorset, UK). Glycine was purchased from Fisher Scientific (Loughborough, UK). Multi-element standard stock solution (10 mg L^−1^ of As, Cd, Cr, Cu, Mn, Ni, Pb and Zn) and Fe standard stock solution (1003 mg L^−1^) were obtained from Qmx Laboratories (Essex, UK).

### Simulation of PM_10_ samples

Samples of PM_10_ were simulated by smearing blank TX40 filters with 100 mg of a soil reference material (BGS RM 102 Ironstone Soil produced by British Geological Survey, Keyworth, UK) using a plastic spatula. This material was selected due to its small particle size (50% of the material by volume is made up of particles <7.6 μm in diameter [34]) and because soil-derived material typically constitutes a major component of airborne PM_10_. Real PM_10_ samples were not used because their mass and composition is variable, whereas it was important to have series of replicate samples (filters loaded with the same mass of a known material) for use in method development. To investigate whether the filters affected the extraction procedure, 100 mg samples of BGS RM 102 Ironstone Soil alone and blank TX40 filters were also extracted.

### Preparation of AMF

To prepare 200 mL of AMF, the inorganic and organic reagents listed in Table 1 were dissolved in 100 mL deionised water in separate 100 mL volumetric flasks. The prepared reagents were then added to a 500-mL HDPE bottle containing the additional reagents. To obtain the desired pH of 7.00 ± 0.20, 200 μL of 37% HCl was added. The bottle was placed on a magnetic stirrer for 3 h before the pH was adjusted to 7.0 ± 0.2 by using 37% HCl or 1 M NaOH.

### Sequential bioaccessibility extraction procedure

Six simulated PM_10_ samples, six BGS RM 102 Ironstone Soil samples, and six blank TX40 filters were placed in 150 mL wide mouth bottles for extraction. In step 1 of the procedure, a 5-mL aliquot of AMF adjusted to pH 7.00 ± 0.20 at 37 ± 2 °C was added to each bottle and briefly shaken manually. The pH of each suspension was checked and where necessary adjusted to the desired value (7.00 ± 0.20) by using solutions of HCl (25, 50, and 100% *v*/*v*) and 1 M NaOH. The bottles were shaken for 1 h at 100 rpm using an end-over-end rotator inside a pre-heated incubator at 37 ± 2 °C. Suspensions were then decanted into 15 mL centrifuge tubes (filters, where present, remained in the extraction vessels) and centrifuged at 4500×*g* for 10 min. A 3.5-mL aliquot of the clear supernatant was removed by means of a pipette, leaving 1.5 mL of AMF, together with the residual soil, in the centrifuge tube. Of the 3.5 mL of extract removed, 2.5 mL was taken and diluted to 10 mL with 2% HNO_3_ in a volumetric flask, then stored at 4 °C for analysis by ICP-MS (see “Chemical analyses” section).

In step 2 of the procedure, either the SBET or the stomach phase of the UBM (as modified in [34]) was applied to the residual soil and AMF. To maintain similar solid/liquid ratios as used in the original protocols (1 g/100 mL for the SBET and 0.6 g/22.5 mL for the stomach phase of the UBM), 10 mL of 0.4 M glycine was added to half of the centrifuge tubes and 2.25 mL of UBM gastric fluid reagent (without addition of saliva fluid since inhalable PM_10_ enter the respiratory tract through the nose) to the other half of the centrifuge tubes. Each centrifuge tube was shaken well and then the contents decanted back into their original 150 mL wide mouth bottle for completion of the sequential extraction procedure.

### Chemical analyses

Extracts obtained were analysed by ICP-MS (Model 7700x, Agilent Technologies, Cheshire, UK). The instrument operating conditions are shown in Table 2. The ICP-MS was calibrated using matrix-matched standard solutions. One of these calibration standards was re-analysed every 10 analyses and also at the end of the sample run to check for instrumental drift. The recovery of the re-analysed standard was in all cases 100 ± 10% of the initial value.

**Table 2:** Operating conditions of the ICP-MS

Power (W)	1550
Quadrupole bias (V)	−15
Octopole bias (V)	−18
Nebulizer gas flow (L min^−1^)	0.85
Plasma gas flow (L min^−1^)	15
Auxiliary gas flow (L min^−1^)	0.9
Isotopes determined	^75^As, ^111^Cd, ^114^Cd, ^52^Cr, ^53^Cr, ^63^Cu, ^65^Cu, ^56^Fe, ^57^Fe, ^55^Mn, ^60^Ni, ^61^Ni, ^206^Pb, ^207^Pb, ^208^Pb, ^64^Zn and ^66^Zn
Isotopes quantified (with internal standards in parenthesis)	^75^As (^72^Ge), ^111^Cd (^115^In), ^52^Cr (^45^Sc), ^63^Cu (^45^Sc), ^56^Fe (^45^Sc), ^55^Mn (^45^Sc), ^60^Ni (^45^Sc), ^208^Pb (^209^Bi) and ^66^Zn (^72^Ge)
Collision cell gas (L min^−1^)	He (4.5) for all masses determined, except for ^111^Cd, ^114^Cd, ^206^Pb, ^207^Pb and ^208^Pb, where no gas mode was chosen
Sample uptake rate (mL min^−1^)	1

### Quality control

Samples were analysed in triplicate (except for AMF where six replicates were used). To check for accuracy, the appropriate extraction reagents were spiked to produce 250 μg L^−1^ concentrations of all PTE tested, except for Fe, where the concentration was 10020 μg L^−1^ and taken through the complete extraction procedure. The percentage spike recovery was calculated by using Eq. 1.

1 $$ \%\kern0.37em \mathrm{spike}\ \mathrm{recovery}=\left(\frac{\left|\mathrm{measured}\ \mathrm{conc}.\mathrm{of}\;\mathrm{PTE}\;\mathrm{in}\;\mathrm{spike}\mathrm{d}\;\mathrm{reagent}-\mathrm{measured}\;\mathrm{conc}.\kern0.37em \mathrm{of}\;\mathrm{PTE}\;\mathrm{in}\;\mathrm{unspiked}\ \mathrm{reagent}\right|}{\mathrm{known}\;\mathrm{conc}.\mathrm{of}\;\mathrm{PTE}\;\mathrm{in}\;\mathrm{spike}\mathrm{d}\;\mathrm{reagent}}\right) \times 100 $$

## Results and discussion

### PTE in blank TX40 FDMS filters

Previous work [34] showed that blank TX40 filters contained significant quantities of Zn, and trace amounts of other analytes, which could be extracted using the SBET or the UBMG (i.e. UBM stomach phase, gastric fluid only) procedures. This was also the case when the AMF was applied, either alone or sequentially with either of the oral bioaccessibility tests (Table 3). A zinc-containing compound is used as a binder in the production of the filters [34] which are standard components of FDMS air monitors. Elimination of the blank either through use of an alternative filter or by washing was not considered appropriate since the goal of the current study was to develop a method applicable to samples obtained as part of conventional air quality monitoring campaigns, where TX40 filters are used as the industry-standard. Instead, all results were corrected for filter blanks for all PTE tested.

**Table 3:** Bioaccessible concentration of potentially toxic elements in blank TX40 filters using artificial mucus fluid (AMF) alone and AMF sequentially with the simplified bioaccessibility extraction test (AMF→SBET) or the stomach phase (gastric fluid only) of the unified bioaccessibility method (AMF→UBMG)

	AMF alone (*n* = 6)		AMF→SBET (*n* = 3)		AMF→UBMG (*n* = 3)	
	μg L^−1^ mean ± SD (RSD)	μg per filter mean ± SD (RSD)	μg L^−1^ mean ± SD (RSD)	μg per filter mean ± SD (RSD)	μg L^−1^ mean ± SD (RSD)	μg per filter mean ± SD (RSD)
As	<0.328	<0.002	<0.017	<0.0002	0.705 ± 0.116 (16.4)	0.003 ± 0.0004 (16.4)
Cd	<0.036	<0.0002	<0.010	<0.0001	0.201 ± 0.057 (28.6)	0.0008 ± 0.0002 (28.6)
Cr	<0.464	<0.002	0.169 ± 0.158 (93.1)	0.002 ± 0.002 (93.1)	13.4 ± 14.8 (111)	0.050 ± 0.056 (111)
Cu	3.63 ± 1.15 (31.8)	0.018 ± 0.006 (31.8)	1.67 ± 2.04 (123)	0.019 ± 0.024 (123)	22.4 ± 2.2 (9.89)	0.084 ± 0.008 (9.89)
Fe	<17.4	<0.087	<20.7	<0.238	<28.5	<0.107
Mn	<0.317	<0.001	4.75 ± 1.89 (40)	0.055 ± 0.022 (40)	43.7 ± 6.0 (13.8)	0.164 ± 0.023 (13.8)
Ni	<0.285	<0.001	0.079 ± 0.078 (99.3)	0.001 ± 0.001 (99.3)	3.59 ± 2.02 (56.4)	0.013 ± 0.008 (56.4)
Pb	<0.359	<0.004	0.383 ± 0.205 (53.5)	0.004 ± 0.002 (53.5)	3.81 ± 0.02 (0.578)	0.014 ± 0.0001 (0.578)
Zn	96.0 ± 47.7 (49.7)	0.480 ± 0.238 (49.7)	537 ± 82 (15.3)	6.18 ± 0.95 (15.3)	2190 ± 215 (9.85)	8.19 ± 0.81 (9.85)

*<* indicates a value less than the instrumental detection limit, *n* number of replicates

### Sequential extraction

#### Step 1: artificial mucus fluid

When simulated PM_10_ samples and samples of soil alone were extracted using the AMF, the bioaccessible PTE concentrations obtained were low, as would be expected due to the neutral pH of the extractant. Only As, Cu, Mn and Ni were detectable (Table 4) with the concentrations of other PTEs less than their respective limits of detection.

**Table 4:** Sequential extraction step 1: bioaccessible concentrations of potentially toxic elements in simulated PM_10_ samples (soil on TX40 FDMS filters) and in soil alone extracted with artificial mucus fluid (*n* = 6)

		As (mg kg^−1^)	Cu (mg kg^−1^)	Mn (mg kg^−1^)	Ni (mg kg^−1^)
Soil	S1	0.742	1.87	6.41	<0.014
	S2	0.805	2.17	6.21	<0.014
	S3	0.792	1.85	6.92	0.178
	S4	0.821	2.00	7.95	0.230
	S5	0.791	1.90	7.27	0.210
	S6	0.761	1.84	8.18	0.211
	Mean	0.785	1.94	7.16	0.207
	SD	0.029	0.13	0.80	0.022
	%RSD	3.68	6.58	11.2	10.6
Soil on TX40 filters	SF1	0.818	1.99	7.52	0.210
	SF2	0.859	2.03	9.96	0.205
	SF3	0.821	1.83	9.81	0.187
	SF4	0.839	1.91	10.6	0.232
	SF5	0.836	1.92	10.9	0.273
	SF6	0.798	1.72	9.78	0.432
	Mean	0.828	1.90	9.76	0.222
	SD	0.021	0.11	1.19	0.033
	%RSD	2.52	5.84	12.2	14.9
%RPD		5.00	2.00	31.0	7.00
Spike recovery		92.3	90.4	92.6	97.1

*SD* standard deviation, *n* number of replicates, *RSD* relative standard deviation, *<* indicates a value less than the procedural limit of detection in AMF, *RPD* relative percent difference = {|a1 − a2|/((a1 + a2)/2)} × 100 where a1 is values in soil alone and a2 is values in soil loaded on TX40 filter. The concentrations of the other analytes fell below their respective limits of detection: Cd 0.018; Cr 0.023; Fe 0.870; Pb 0.018; Zn 0.042 (all mg kg^−1^) and so no data are reported

Statistical results obtained (*t* test at 0.05 significance level) and the calculation of the RPD showed that there was no significant difference between the bioaccessible concentration of PTE in soils alone and in soil on TX40 filters, except for Mn, where larger amounts were recovered from the samples on filters. A combination of small-scale sample heterogeneity and use of a low sample mass (100 mg) may have enhanced variability for this analyte.

#### Step 2: artificial stomach fluid

Higher analyte concentrations were obtained when the UBMG procedure was used as the second step in the sequential extraction than when the SBET was used, except for Fe (Table 5). Since the first step of the sequential extraction is identical, this is probably due to the different pH values used in the SBET (pH 1.5) and UBMG (pH 1.2). For Fe, the lower extractability using the UBMG may be due to increased formation of insoluble precipitates [42] caused by the larger phosphate concentration present. Both of the step 2 extracts contained phosphate derived from the 1.5 mL of AMF carried forward from step 1 of the sequential extraction, but the AMF→UBMG sequence involved addition of 2.25 mL of the UBMG reagent (increasing the phosphate concentration to 208 mg/L) whereas the SBET reagent used in the AMF→SBET sequence contained no additional phosphate.

**Table 5:** Sequential extraction step 2: bioaccessible concentrations of potentially toxic elements in simulated PM_10_ samples (soil on TX40 FDMS filters) and in soil alone extracted using either the simplified bioaccessibility extraction test (SBET) or the stomach phase of the unified bioaccessibility method (gastric fluid only) (UBMG), following extraction with artificial mucus fluid in step 1 (see Table 4)

	Sample								%Spike recovery		%RPD	
	Soil				Soil on TX40 filters							
	SBET		UBMG		SBET		UBMG		SBET	UBMG	SBET	UBMG
	Mean ± SD (mg kg^−1^) (*n* = 3)	%RSD	Mean ± SD (mg kg^−1^) (*n* = 3)	%RSD	Mean ± SD (mg kg^−1^) (*n* = 3)	%RSD	Mean ± SD (mg kg^−1^) (*n* = 3)	%RSD				
As	3.09 ± 0.09	2.85	3.54 ± 0.08	2.22	2.98 ± 0.07	2.37	3.38 ± 0.07	2.18	94.4	95.6	3.56	4.59
Cd	0.149 ± 0.005	3.42	0.224 ± 0.013	5.71	0.145 ± 0.003	2.33	0.219 ± 0.021	9.40	90.9	97.2	2.10	2.17
Cr	30.8 ± 0.8	2.50	39.3 ± 1.4	3.43	30.5 ± 0.2	0.784	33.9 ± 2.1	6.07	88.0	97.3	1.11	14.6
Cu	6.02 ± 0.09	1.41	7.47 ± 0.45	6.05	6.36 ± 0.08	1.28	7.11 ± 0.80	11.3	90.5	96.8	5.48	4.91
Fe	1040 ± 13	1.25	903 ± 55	6.13	1030 ± 14	1.39	842 ± 26	3.11	72.6	70.7	0.321	6.94
Mn	1840 ± 29	1.58	2710 ± 113	4.16	1800 ± 26	1.45	2600 ± 144	5.57	89.2	108	2.35	4.40
Ni	7.97 ± 0.07	0.933	11.4 ± 0.5	3.99	7.79 ± 0.15	1.94	10.4 ± 0.3	3.11	90.1	94.1	2.34	9.60
Pb	18.4 ± 0.3	1.59	23.1 ± 1.1	4.71	17.8 ± 0.3	1.79	21.6 ± 1.1	4.91	85.0	101	3.15	6.77
Zn	28.9 ± 0.7	2.49	40.3 ± 2.5	6.26	21.9 ± 2.9	13.3	30.9 ± 1.8	5.73	86.2	107	27.5	26.5

*SD* standard deviation, *n* number of replicates, *RSD* relative standard deviation, *RPD* relative percent difference = {|a1 − a2|/((a1 + a2)/2)} × 100 where a1 is values in soil alone and a2 is values in soil loaded on TX40 filter

The RPD between the bioaccessible concentrations of PTE obtained in soils alone and in soils on TX40 filters were <10% for the majority of analytes confirming that the presence of the filter did not affect the extraction efficiency, so long as blank correction was performed. The exception was Zn, where higher results were obtained for soil alone than for soil on TX40 filters. It is possible that variability between the Zn content in filters may have led to overcorrection of the filter blank. This highlights the difficulties of determining bioaccessible Zn accurately when the industry-standard TX40 filter is used.

### Comparison between sequential and single extraction

**Fig. 1:**
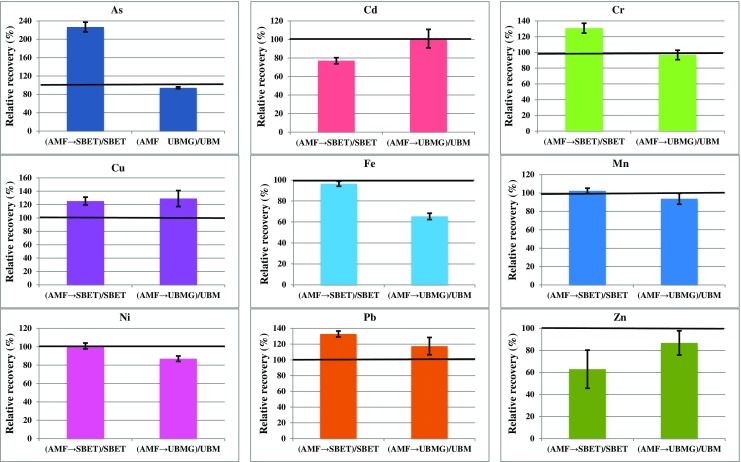
Comparison between the bioaccessible concentrations of potentially toxic elements obtained by sequential extraction and by use of an oral bioaccessibility test alone for simulated PM_10_ samples (soil on TX40 FDMS filters). Results are expressed as the relative recovery of the sequential procedures relative to their corresponding single-step oral bioaccessibility test, where *AMF→SBET* = sequential extraction with artificial mucus fluid followed by the simplified bioaccessibility extraction test; *AMF→UBMG* = sequential extraction with artificial mucus fluid followed by the stomach phase of the unified bioaccessibility method (gastric fluid only); *SBET* = simplified bioaccessibility extraction test alone; *UBM* = stomach phase of the unified bioaccessibility method alone. *Error bar* represents 1 standard deviation, *n* = 3. Data for the SBET and UBM procedures alone from reference [34]

Comparing the total amounts of PTE extracted from simulated PM_10_ samples by the two-step sequential procedures developed—either AMF followed by SBET (AMF→SBET) or AMF followed by UBMG (AMF→UBMG)—and the amounts of PTE extracted when the corresponding oral bioaccessibility tests (SBET or UBM stomach phase) were applied alone [34] revealed some interesting trends (Fig. 1). Whilst the amounts of analyte released by single and sequential extraction were in many cases similar (i.e. relative recovery values were close to 100%), the sequential AMF→SBET method released substantially more As, Cr, Cu and Pb than the SBET alone, whilst the sequential AMF→UBMG released more Cu and Pb than the UBMG alone. Without the addition of the mucus phase to create a more physiologically relevant extraction environment, the bioaccessible concentrations of these elements would therefore have been underestimated.

Statistical analysis (*t* test at 0.05 significance level) (see Table 6) indicated that bioaccessible PTE concentrations were significantly different between the single and sequential extraction procedures for As, Cd, Cr, Cu and Pb using the SBET-based procedures, and for As, Cu, Fe, Ni and Pb using the UBM-based procedures. Analytes could be grouped into three categories for each method. The first group showed no significant difference between single and sequential extraction; the second gave higher bioaccessible analytes concentrations using the sequential extraction, whilst the third gave higher bioaccessible analyte concentration using the oral bioaccessibility test alone. For the SBET, the first group comprised Fe, Mn, Ni and Zn; the second As (+127%), Cr (+31%), Cu (+25%) and Pb (+33%); and only Cd (−23%) was present in the third group. For the UBM, the first group included Cd, Cr, Mn and Zn; the second group Cu (+29%) and Pb (+17%); and the third As (−6%), Fe (−35%) and Ni (−13%).

**Table 6:** Statistical comparison between the bioaccessible concentrations of potentially toxic elements obtained by sequential extraction and by use of an oral bioaccessibility test alone for simulated PM_10_ samples (soil on TX40 FDMS filters)

PTE	SBET vs. AMF→SBET (*ν* = 4)	UBM (stomach phase) vs. AMF→UBM (*ν* = 4)
	*t* calculated	*t* calculated
As	*37.44*	*4.19*
Cd	*9.78*	0.16
Cr	*11.08*	0.91
Cu	*9.60*	*4.30*
Fe	2.48	*14.92*
Mn	1.44	1.96
Ni	0.48	*6.88*
Pb	*16.95*	*3.20*
Zn	2.52	1.93

*ν* degree of freedom, significance level (*α*) = 0.05, *t* critical for all cases = 2.78, *AMF→SBET* sequential extraction with artificial mucus fluid followed by the simplified bioaccessibility extraction test, *AMF→UBMG* sequential extraction with artificial mucus fluid followed by the stomach phase of the unified bioaccessibility method (gastric fluid only), *SBET* simplified bioaccessibility extraction test alone, *UBM* stomach phase of the unified bioaccessibility method alone. Italics indicates values that failed the *t *test

The difference in solubility of PTE between the single and the sequential procedures is due to interaction between a number of factors. One of most important is pH. The addition of 1.5 mL residual AMF to the step 2 reagent increases the pH from the typical values of 1.5 (SBET) or 1.2 (UBMG) and this may be responsible for the decreased recoveries of Cd for the AMF→SBET sequential extraction relative to the SBET, and of Fe and Ni for the AMF→UBMG sequential extraction, relative to the stomach phase of the UBM. The composition of the extractants is also influential, especially the exchangeable ion and complexing ligand contents. For the AMF→SBET sequential extraction, enhanced release of As and Cr may have been due to the presence of NaH_2_PO_4_ in the step 1 reagent. Ion exchange reactions between PO_4_^3−^ in solution and oxyanions (AsO_3_^3−^, AsO_4_^3−^ and CrO_4_^2−^) at the edges of soil particles can increase analyte solubility [42]. This was not observed for the AMF→UBMG sequence because the UBMG reagent already contained phosphate. The greater amounts of Cu and Pb extracted when either AMF→SBET or AMF→UBMG was applied may be due to the presence of larger numbers and amounts of complexing organic acids in the sequential extraction reagents than in the SBET or UBM (stomach phase) alone. Both these elements have a high affinity for organic acid ligands [43].

### Quality control

The results obtained show that the spike recoveries of PTE (see Tables 4 and 5) in all extraction procedures conducted in this work were between 85 and 114% except for Fe, where it was 72.6 and 70.7% for SBET2 and UBMG, respectively, when these were conducted sequentially after the extraction by the AMF. This may be due to the formation of insoluble phosphates of Fe [42]. For the precision expressed as relative standard deviation (% RSD), approximately 87% of the RSD values were <10, and 13% of the values were between 10 and <15% (see Tables 4 and 5).

## Conclusion

In this work, a novel artificial AMF was designed for use as the first step of a sequential extraction, in combination with either the SBET or the UBMG oral bioaccessibility test, to better simulate physiological conditions experienced by PM transported into the gastrointestinal tract by mucociliary clearance and thus provide a more accurate estimation of the human bioaccessibility of PM-bound PTE following inhalation. Methods were developed successfully using simulated PM samples composed of a soil reference material supported on TX40 filters used in FDMS systems for continuous measurement of airborne particulate matter. Analysis of blank TX40 FDMS filters revealed that bioaccessible concentration of all PTE tested was low, except for Zn, where a significant blank concentration was found. Different amounts of analytes (except Mn and Zn) were recovered using the sequential extraction procedures (AMF→SBET and AMF→UBMG) than when the respective SBET or UBM (stomach phase) were applied alone. Both sequential extraction procedures isolated greater amounts of Cu and Pb than the equivalent oral bioaccessibility tests. The AMF→SBET procedure also recovered higher quantities of Cr and, especially As (more than twice the amount released by the SBET procedure alone). The study demonstrated that, to assess the bioaccessibility of PTE associated with inhaled PM, a sequential extraction test involving AMF followed by artificial gastric fluid (either the SBET or the UBM) should be adopted, otherwise the bioaccessible concentration of trace elements with potentially serious impact on human health may be estimated incorrectly.
